# Supplementary material for: Identifying Anthropogenic Sources of Heavy Metals in Alpine Peatlands over the Past 150 Years: Examples from Typical Peatlands in Altay Mountains, Northwest China
Source: Int J Environ Res Public Health. 2023 Mar 12;20(6):5013. doi: 10.3390/ijerph20065013 (PMC10048823; doi:10.3390/ijerph20065013)
Supplement: Supplementary file 1 [file ijerph-20-05013-s001.zip › ijerph-2244921-supplementary.pdf]

Table S1. Overview of typical Peatlands profiles in the Altay Mountains.

Sampling Site	Altitude (m)	Peat Profile Characteristics
Jiadengyu (JDY)	1402	The thickness of the profile is 60 cm; from top to bottom it can be divided as follows: turf layer: 0–7 cm, vegetation cover is about 80%. Grass roots are dense, containing green plant bodies; humus layer: 7–17 cm, poor decomposition, mainly incompletely decomposed mosses and mosses, dense modern plant roots, and dark brown loamy soil with more roots; peat layer: dark brown, containing more capillary roots; 17–21 cm, black brown powdery clay with less roots; 21–30 cm brown clay, basically no roots below 30 cm.
Heihu (HH)	2190	The thickness of the profile is 30 cm; from top to bottom it can be divided into: turf layer: 0–5 cm, vegetation cover is about 80%. Grass roots are dense, containing green plant bodies; humus layer: 5–15 cm, poorly decomposed, mainly incompletely decomposed mosses and mosses, dense modern plant roots, and dark brown loamy soil with more roots; peat layer: dark brown, loamy clay to clay, blocky, containing more capillary roots; 15–30 cm is brown peat layer.

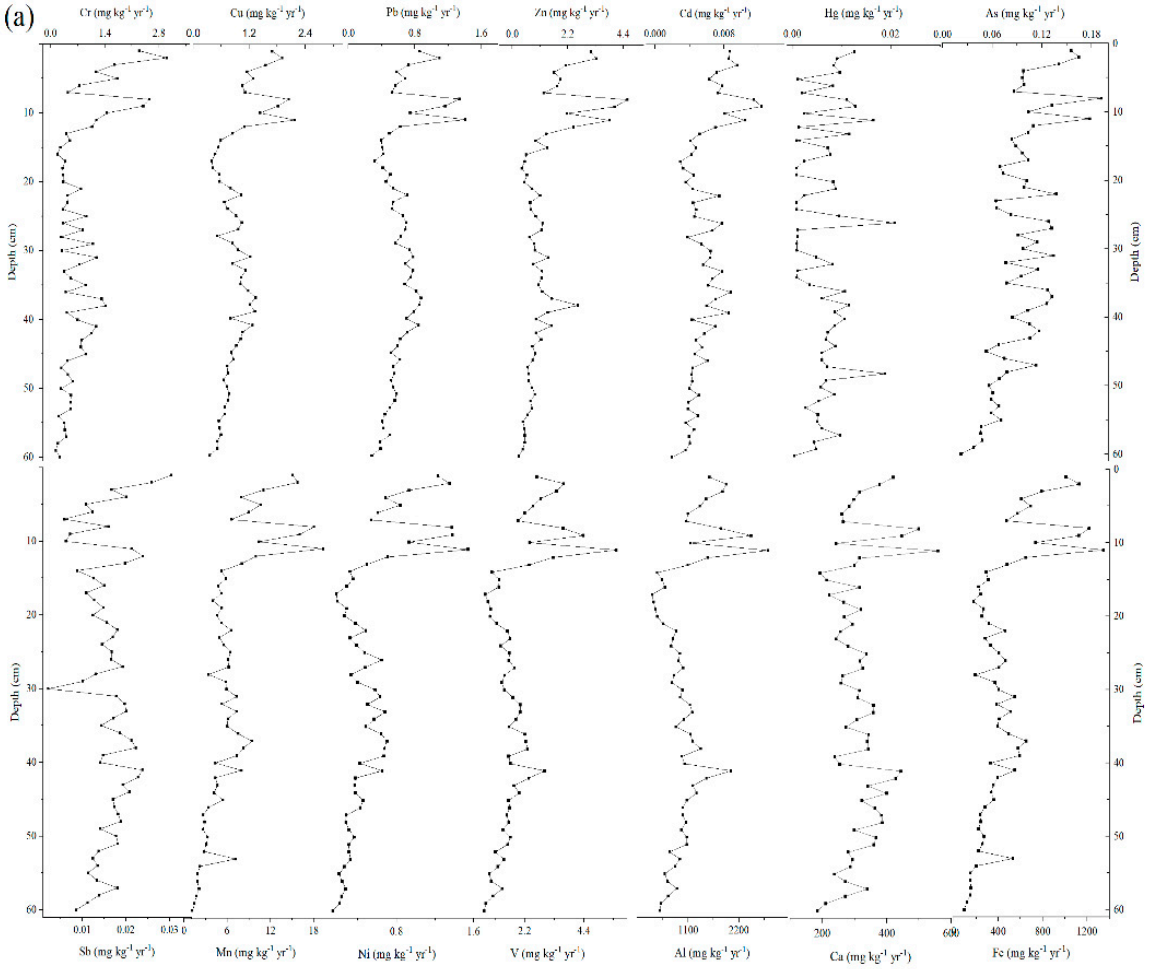

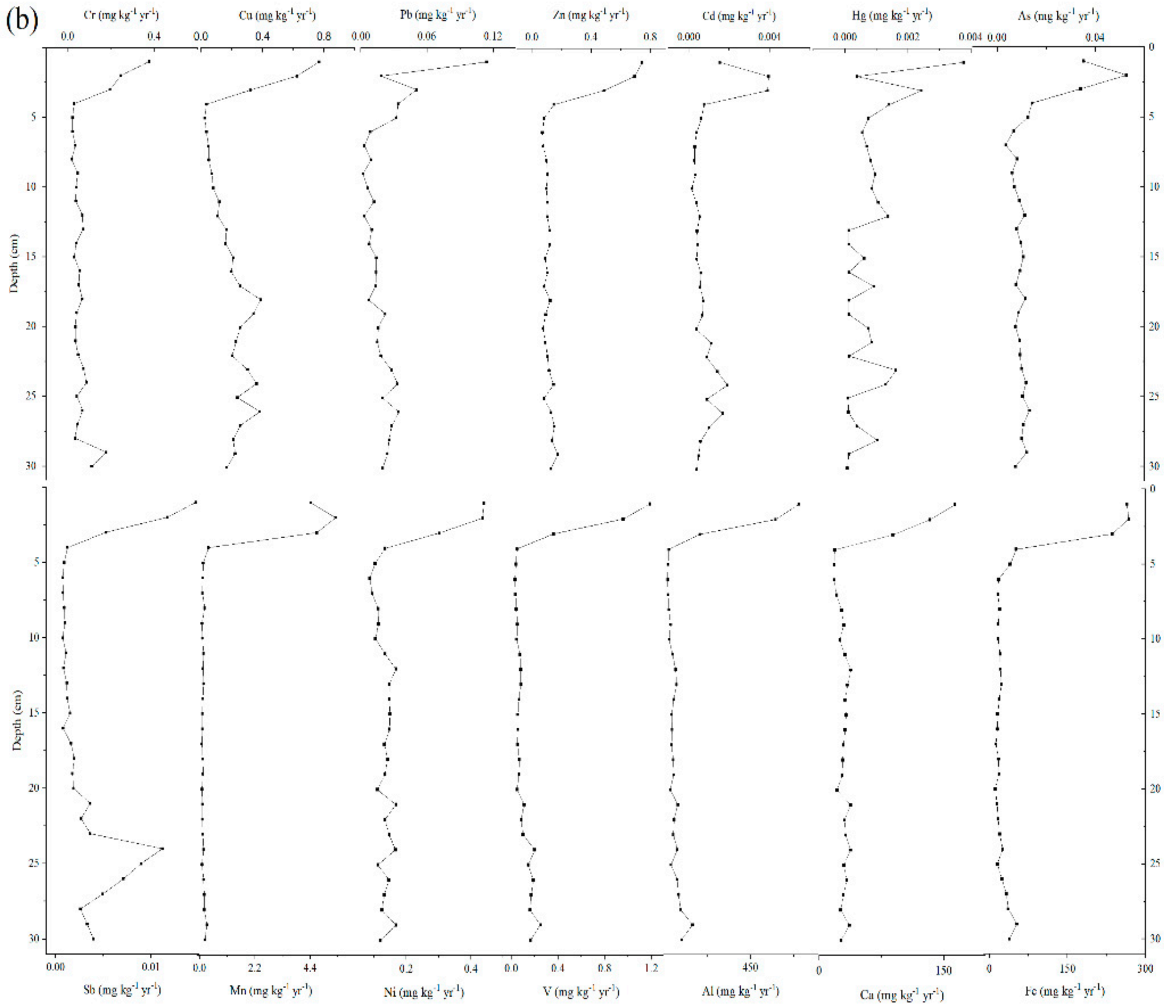

Figure S1. Profiles of accumulation rate of pollution elements for JDY(**a**) and HH(**b**) peatlands in Altay Mountain.

Text S1：PCA (principal component analysis) characteristics in JDY peatlands

The principal component analysis (PCA) was used to analyze the source of HMs in peat, and the suitability of PCA for the data set was analyzed by KMO and Bartlett's test, yielding a KMO value of 0.795. In the Bartlett's sphericity test, the approximate chi-square value of 45 degrees of freedom was 1126.748 (df = 105, $p < 0.01$); it indicates that there is a strong correlation between the HMs. It is generally accepted that KMO > 0.7 is suitable for factor analysis. The combinations of HMs in peat bog were classified by factor analysis and other data reduction techniques. The PCA in the JDY core is shown in Table S2. The 15 components were grouped into four principal factors with a cumulative total variance contribution of 84.311% after rotation by maximum orthogonal method. The first principal factor (F 1) had the largest loadings of Fe, Zn, Mn, Ti, Ni, Cr, and V, with a variance contribution of 46.165% and an eigenvalue of 6.925. The second principal factor (F 2) had larger loadings of Sb, Ca, Hg, and Al, with a variance contribution of 24.861% and an eigenvalue of 3.729. The third principal factor (F 3) had the largest loadings of Cd, Pb, and Cu and had the highest loading with a variance contribution rate of 7.067% and eigenvalue of 1.06. The fourth principal factor (F 4) had a higher loading of As with a variance contribution rate of 6.218% and eigenvalue of 0.933.

Table S2. Loadings of HMs on VARIMAX-rotated factors of different datasets in JDY peatlands.

HMs	Factor Loading			
	F1	F2	F3	F4
Fe	0.903	-0.176	0.226	0.147
Zn	0.886	-0.112	0.193	0.141
Mn	0.878	-0.344	0.007	0.126
Ti	0.867	0.285	0.098	-0.094
Ni	0.846	-0.143	0.422	0.034
Cr	0.788	0.022	0.067	0.048
V	0.748	0.478	0.320	-0.117
Sb	-0.125	0.887	0.132	-0.036
Ca	-0.234	0.821	0.371	-0.129
Hg	0.004	0.695	0.033	0.293
Al	0.519	0.650	0.464	-0.128
Cd	0.220	0.138	0.863	0.108
Pb	0.126	0.503	0.735	-0.103
Cu	0.580	0.227	0.730	0.089
As	0.126	0.050	0.049	0.937
Eigenvalues	6.925	3.729	1.06	0.933
% Variance	46.165	24.861	7.067	6.218
% Cumulative variance	46.165	71.026	78.093	84.311

Text S2：PCA (principal component analysis) characteristics in HH peatlands

The PCA in the HH peat core is shown in Table S4. The 15 components were grouped into four principal factors with a cumulative total variance contribution of 81.615%. The first principal factor (F 1) had the largest loadings of Ti, Al, Zn, V, Mn, Fe, Ca, and Cr with a variance contribution rate of 44.59% and an eigenvalue of 6.688, while the second principal factor (F 2) had larger loadings of Pb, Cd, As, and Sb with a variance contribution rate of 16.468% and an eigenvalue of 2.47. The third principal factor (F 3) had the largest loading of Ni. The fourth principal factor (F 4) had a higher loading of Hg and Cu with a variance contribution of 6.435% and an eigenvalue of 0.965.

Table S3. Loadings of HMs on VARIMAX-rotated factors of different datasets in HH peatlands.

HMs	Factor Loading			
	F1	F2	F3	F4
Ti	0.930	0.070	0.234	0.117
Al	0.929	0.079	0.172	0.116
Zn	0.888	0.269	-0.074	-0.197
V	0.855	0.338	0.288	0.168
Mn	0.833	0.236	-0.421	-0.083
Fe	0.814	0.3047	-0.303	-0.283
Ca	0.807	-0.054	0.062	0.119
Cr	0.702	-0.005	0.342	0.168
Pb	0.073	0.824	0.250	-0.242
Cd	0.131	0.816	-0.175	0.259
As	0.329	0.688	-0.464	-0.101
Sb	0.154	0.647	0.425	0.409
Ni	0.259	0.032	0.775	0.141
Hg	-0.076	0.113	-0.012	-0.837
Cu	0.005	0.358	0.336	0.705
Eigenvalues	6.688	2.47	2.118	0.965
% Variance	44.590	16.468	14.123	6.435
% Cumulative variance	44.590	61.058	75.181	81.615

Table S4. Elemental mean and background values.

Elements	Mean Value in JDY	Mean Value in HH	Background Value of Soil Elements
Ti	1507.18	980.59	3117
Cu	37.60	66.17	31.02
Pb	25.57	6.21	23.63
As	3.75	3.53	11.2
Cd	0.23	0.26	0.12
Zn	46.17	43.51	99.2
Cr	28.49	20.01	58.63
Ni	19.62	39.92	47.16
Hg	0.26	0.23	0.02
Mn	237.27	106.73	907.6
V	72.86	38.74	100
Fe	16449.31	10804.14	30890
Sb	0.67	0.96	0.31
Al	41984.00	22046.03	77440
Ca	13311.13	10774.29	29450

Text S3：Correlation Analysis

The relationship between HMs can reflect their sources and migration, and the correlation between HMs contents was significant or highly significant, indicating some homologous relationship or compound contamination, and if they are not interrelated, then it means that HMs are not controlled by a single factor. In order to determine the sources of HMs in the peat of JDY (Figure 8a), Pearson correlation analysis was used to analyze the HMs contents of the peat sediment. The results showed that except for the negative correlation between Sb and elements, the correlation between the other elements was mainly positive and reached 0.05 significance levels, indicating that the elements have similar sources. HH peat showed that the correlations among the elements were mainly positive (Figure 8b); Cu showed high correlations with elements Cd and Sb at 0.05 and 0.01 levels of significance, indicating similarity in origin; Pb showed significant positive correlations with elements Cd, As, V, Sb, and Fe. The correlation of Cr was obtained with Mn, V, Al, Ca, Ti, and Fe at the 0.01 significance level. The correlations between Mn and Cd, Zn, Cr, V, Al, Ca, Ti, and Fe were significant and positive, and the correlations between V, Sb, Al, Ca, and Ti were more consistent.

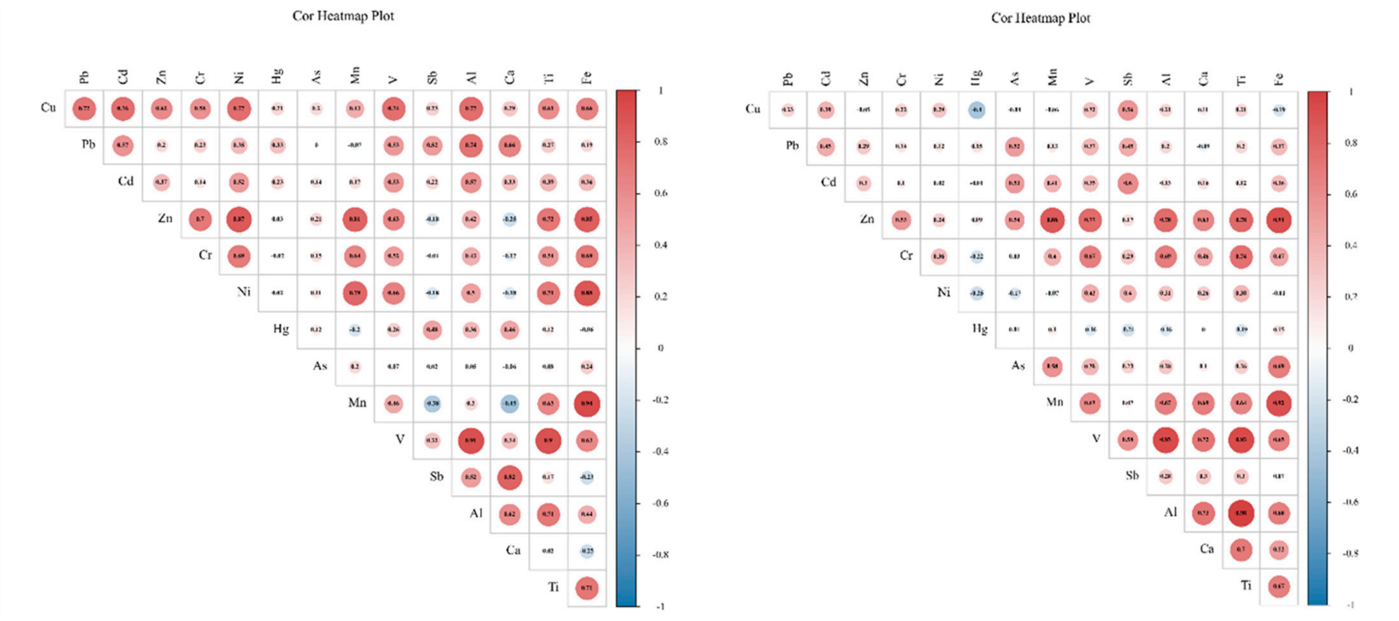

Figure S2. Correlation between HMs contents in peat sediments of JDY and HH.
